# Editorial: New approaches for the prevention and reduction of metabolic disease in menopausal women

**DOI:** 10.3389/fnut.2024.1359853

**Published:** 2024-03-28

**Authors:** Seong-Hee Ko

**Affiliations:** Department of Food Science and Nutrition, Sookmyung Women's University, Seoul, Republic of Korea

**Keywords:** postmenopausal women, metabolic disease, osteoporosis, cardiovascular disease, bone mineral density

Menopause is a natural aging process that occurs in all women and is clinically diagnosed after loss of follicular activity in the ovaries, resulting in amenorrhea 12 months after the last menstrual period. Menopause typically occurs between the ages of 45 and 55, with an average age of 51. Women generally live longer than men, and as life expectancy increases globally, life expectancy for women is also increasing. If women live to age 90, they spend almost half their lives in menopause. Menopause occurs over several years, and women experience irregular menstrual cycles called the menopausal transition or perimenopause, accompanied by a cessation of oocyte production by the ovaries. One of the biggest physiological changes in menopausal women is the secretion of estrogen, a sharp decrease in which determines the physiological characteristics of a woman. Both natural menopause and surgically induced menopause (due to removal of the ovaries) are accompanied by rapid hormonal changes, which can lead to cardiovascular disease and metabolic syndrome, including type 2 diabetes. The resulting dysregulation of lipid metabolism affects various aspects of energy metabolism, such as body fat mass, fat-free mass, fatty acid metabolism, basal metabolic rate, and obesity.

Particularly, estrogen deficiency can lead to chronic inflammatory conditions, including increased pro-inflammatory cytokines, impaired Treg/Th17 cell balance, and impaired osteogenic differentiation capacity of bone marrow mesenchymal stem cells (BMSCs). Inadequate bone formation by BMSC-derived osteoblasts to compensate for bone resorption by osteoblasts is a major cause of osteoporosis in postmenopausal women. In addition to this, menopause is associated with pathological menopausal syndromes, such as disturbances in sleep/mood, vasomotor symptoms (including hot flashes and night sweats), urogenital atrophy, osteopenia and osteoporosis, psychiatric disorders, sexual dysfunction, skin lesions, cardiovascular diseases (CVDs), cancer, metabolic disorders, and obesity. Women are at a higher risk of developing CVDs after menopause due to estrogen deficiency and dysregulated lipid metabolism.

This Research Topic is aimed at collecting articles suitable to improve our knowledge and understanding on beneficial nutrients and food-derived compounds for metabolic disorder in postmenopausal women, postmenopausal osteoporosis, bone remodeling, bone metabolic abnormalities. Moreover, this Research Topic also covers the beneficial effects of diets and nutrients on menopausal symptoms, e.g., vasomotor symptoms, cognitive function, and quality of life in postmenopausal women, and dietary factors and the reduction of disease risk in postmenopausal women (including cardiovascular disease, osteoporosis, and breast cancer).

This Research Topic has four articles covering the aspects mentioned above.

Friling et al. reported a study comparing the calcium bioavailability of postbiotics calcium-carrying *Lactobacillus* and calcium-carrying *Saccharomyces cerevisiae* with calcium citrate in postmenopausal women aged 45–65 years. The postmenopausal female population is at higher risk of losing bone mass and developing osteoporosis due to the significant decline in estradiol (E2) associated with menopause. Therefore, appropriate calcium supplementation is recommended to prevent losing bone mass and developing osteoporosis. In this situation, the research by Friling et al. on highly bioavailable calcium supplements suggests that the postbiotic calcium-carrying *Lactobacillus* may be a good source of calcium for postmenopausal women.

The role of hypertension in bone mineral density (BMD) in men over 50 years of age and postmenopausal women was reported by the Li et al.. It is based on evidence from the 2005–2010 National Health and Nutrition Examination Survey. Hypertension was defined as participants with mean systolic blood pressure (SBP) ≥140 mmHg, mean diastolic blood pressure (DBP) ≥90 mmHg, or taking medications prescribed for hypertension, and bone mineral density values at the femoral neck and lumbar spine were measured as the primary outcome. It was reported that there was a positive association between hypertension and lumbar BMD and the lumbar BMD was significantly higher in the presence of hypertension than in the control group in both males (1.072 vs. 1.047 g/cm^2^) and females (0.967 vs. 0.938 g/cm^2^; both *p* < 0.05), but a similar pattern was not found in the femoral neck. Meanwhile, lumbar BMD was positively associated with SBP and negatively associated with DBP both in males and females. In other words, this study suggests that hypertension is associated with higher bone mineral density in the lumbar spine in both men over 50 years of age and postmenopausal women. Although the results of these epidemiological studies may give rise to much controversy, these studies will contribute as a basis for analyzing the relationship between high blood pressure and bone mineral density in postmenopausal women.

Interestingly, for the first time, the efficacy and synergistic effects of Kyung-Ok-Ko (KOK) and *Pueraria lobata Ohwi* (*P. lobata*) in alleviating postmenopausal symptoms, particularly focusing on osteoporosis, were reported through a comprehensive *in vivo* study using ovariectomized (OVX) rats. KOK is a traditional oriental medicine consisting of four ingredients: *Panax ginseng, Wolfiporia extensa, Rehmannia glutinosa*, and honey (Kim et al.). In East Asia, KOK has long been administered as a vitalizing medicine for healthy people, or with medicinal intent to treat patients with various age-related symptoms. This study explores the potential of KOK in its mixture with *P. lobata* as an alternative treatment for menopausal symptoms, and its effects on bone health and the regulation of autophagy-related kinases are reported. Uterine morphology, estrogen receptor expression, and liver protein expression were also assessed. In particular, the protective effect of the treatment against hyperlipidemia and osteoporosis was verified through BMD and serum osteocalcin measurements. Such research may help us understand the bidirectional relationship between menopausal osteoporosis and these phytoestrogens and expand the scope of research on alleviating menopausal symptoms.

Generally, menopause is a period of physiological and psychological changes that increase the risk of cardiovascular disease in part due to the increased prevalence of metabolic syndrome (MetS). Recently, Silva et al. (1) reported a narrative review on the current evidence on the association between dietary patterns and clinical endpoints in postmenopausal women, such as body composition, bone mass, and risk markers for cardiovascular disease. Current evidence suggests that low-fat plant-based diets have beneficial effects on body composition. However, further studies are needed to confirm these findings in postmenopausal women. It has been reported that the Mediterranean dietary pattern, along with other healthy habits, may help in the primary prevention of bone, metabolic, and cardiovascular diseases after menopause, which consists of the use of healthy foods with anti-inflammatory and antioxidant properties, which are associated with small but significant reductions in blood pressure, reduced fat mass and improved cholesterol levels. These effects must be assessed over a long period of time through assessment of difficult outcomes such as fractures, diabetes, and coronary ischemia, requiring long-term study planning and ongoing monitoring. Some included the Mediterranean or Dietary Approaches to Stop Hypertension (DASH) diets as part of the intervention regimen. However, there is a significant paucity of RCTs testing the Mediterranean or DASH diets, despite the wider literature suggesting that the Mediterranean diet in particular is effective for reducing postmenopausal MetS. Therefore, it is important to accumulate information on research design and interventions through systematic literature reviews.

In summary, the studies and reviews covered above contain the most up-to-date information regarding metabolic diseases in postmenopausal women. Extensive epidemiological studies, animal experiments, systematic literature reviews, and human experiments reflecting hormonal changes in postmenopausal women provide important information for preventing and treating osteoporosis, hypertension, and various metabolic diseases in postmenopausal women. In addition, it may open a new chapter in the field of future research design and the discovery of bioactive substances.

## Author contributions

S-HK: Writing – original draft, Writing – review & editing.
